# Can Aluminum Tolerant Wheat Cultivar Perform Better under Phosphate Deficient Conditions?

**DOI:** 10.3390/ijms19102964

**Published:** 2018-09-28

**Authors:** Mohammad Rezaul Karim, Xiaoying Dong, Lu Zheng, Renfang Shen, Ping Lan

**Affiliations:** 1 State Key Laboratory of Soil and Sustainable Agriculture, Institute of Soil Science, Chinese Academy of Sciences, Nanjing 210008, China; rezaul.karim@issas.ac.cn (M.R.K.); xydong@issas.ac.cn (X.D.); luzheng@issas.ac.cn (L.Z.); rfshen@issas.ac.cn (R.S.); 2 University of Chinese Academy of Sciences, Beijing 100049, China

**Keywords:** phosphate deficiency, wheat, Al-tolerance, gene expression

## Abstract

Low availability of inorganic phosphate (Pi), together with aluminum (Al), is a major constraint for plant growth and development in acidic soils. To investigate whether or not Al-resistant cultivars can perform better under Pi deficiency, we chose two wheat cultivars with different Al-responses—Atlas 66, being Al-tolerant, and Scout 66, which is Al-sensitive—and analyzed their responses to Pi deficiency. Results showed that, unexpectedly, the Al-sensitive cultivar Scout 66 contained comparatively higher amount of soluble phosphate (Pi) and total phosphorus (P) both in the roots and in the shoots than Atlas 66 under P deficiency. In addition, Scout 66 exhibited higher root biomass, root volume, and root tip numbers, compared with Atlas 66. The expression of Pi-responsive marker genes, *TaIPS1*, *TaSPX3*, and *TaSQD2* was strongly induced in both cultivars, but the extents of induction were higher in Scout 66 than in Atlas 66 under long-term Pi starvation. Taken together, our results suggest that the Al-sensitive cultivar Scout 66 performed much better under sole Pi starvation, which sets the following experimental stage to uncover the underlying mechanisms of why Scout 66 can display better under Pi deficiency. Our study also raises an open question whether Al-resistant plants are more sensitive to Pi deficiency.

## 1. Introduction

Phosphorus (P) is an essential macronutrient for the growth and development in plants. The total P is naturally abundant in soil; however, the plant available P, inorganic phosphate (Pi), is always beyond the demand of growth in natural and agricultural ecosystem, especially in acidic and calcareous soils. The low bio-availability of P is due to the rapid transformation of Pi into organic P and the formation of insoluble Pi complexes with soil cations, such as aluminum (Al) and iron (Fe) or calcium and manganese under acidic or alkaline soils, respectively [1,2]. A large amount of P-fertilizers over the crop’s demand therefore are normally supplied to maintain or increase the crop yields, which results in the future non-renewable P source scarcity and severe environmental concerns due to the run-off of excess Pi [3,4,5]. Therefore, it is clear that excess use of P-fertilizers is neither sustainable nor environmental friendly. Hence, to understand how plants respond to P deficiency and translate the gained knowledge to improve P-acquisition and P-use efficiency is a major approach to maintain food security and reduce environmental damage.

To cope with low Pi stress, plants have evolved an array of adaptive processes to improve Pi uptake and remobilization when the external Pi pool is limited. Such responses include morphological and physiological changes, as well as biochemical and molecular regulation [6,7,8,9,10,11]. The morphological changes include the increase of root length in monocot [12], an increase of root biomass and root-shoot ratio, and the final alteration of root system architecture [13,14]. Biochemical adaptations comprise the increased excretion of organic acids and increased secretion of Pi-releasing enzymes, such as RNases and purple acid phosphatases, providing the roots a larger rhizospheric Pi pool for uptake [15,16,17]. Along with the increased Pi pools, the expression and activity of high-affinity Pi-transporters, controlling the influx of extracellular Pi, are induced [2]. Besides Pi-transporter, omics approaches have revealed that more than 1000 genes and hundreds of proteins are regulated by Pi [2,11,18]. Some major controllers, such as *PHR1*, *SPX1*, *SPX3*, *SIZ1* and so on from *Arabidopsis thaliana* and homologies from rice and other species have been documented to play central roles in the regulation of gene activity [19,20,21,22,23,24,25,26,27,28,29,30,31,32,33,34,35]. The sensor for Pi until recent was reported [36].

While the Pi deficiency responses have been expansively documented in the model plant Arabidopsis, limited information is available of the responses to Pi deficiency in agronomically important crops, such as wheat [6]. Wheat is one of the major staple food crops in the world in terms of both the cultivation area and the consumption and prevalence as a food source. A quite lot of wheat cultivation area is in the tropical and subtropical regions, with the arable soils in these regions being acidic with low P level, which is a major limiting factor for wheat production. It is estimated that ~40% of the potentially arable lands are acidic in the world. In Acidic soils, with the decrease of pH below 5, non-toxic Al is solubilized into Al ion, a form of extremely toxic to plant growth [37]. Meanwhile, with the release of ions of Al and Fe, most Pi become insoluble complexes with these cations. Thus, Al toxicity (and possible Fe toxicity) and Pi deficiency occur simultaneously in acidic soils [37,38,39], both of which cause severe losses of wheat yields. It is therefore becoming urgent to find a solution to develop Pi-efficient wheat cultivars for sustainable wheat production [6].

The two wheat cultivars Atlas 66 and Scout 66 are well known for their contrasting behavior in Al toxicity conditions (Atlas 66 is Al-tolerant, while Scout 66 is Al-sensitive) and have been extensively studied regarding Al responses [40,41]. In this study, the responses of these two cultivars to sole Pi deficiency were observed and results showed that Scout 66 performed much better under sole Pi starvation than Atlas 66. This study raises two open questions for future attempts: (1) Whether Al-resistant plants are more sensitive to Pi deficiency; (2) how plants balance the responses to different stresses simultaneously.

## 2. Results

### 2.1. Scout 66 Showed Higher Root and Shoot Biomass under Phosphate Starvation

**Figure 1:**
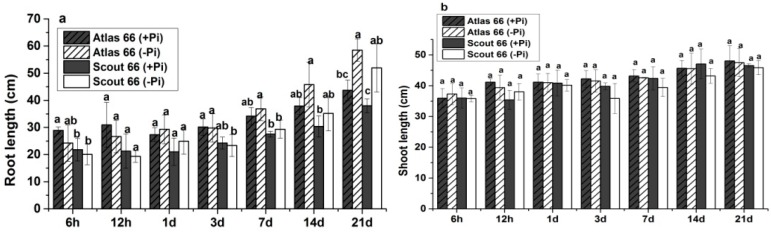
Root length (**a**), and shoot length (**b**) of two wheat cultivars in different observed time points (6 h to 21 d) in phosphate sufficient (250 µM) and deficient (0 µM) conditions. Error bars are Standard Deviation (SD), *n* = 6. Different small letters indicated significant differences (*p* ≤ 0.05) among the different treatment combinations within the same time point.

To comparatively explore the physiological responses of the two cultivars to Pi deficiency, three-week-old seedlings grown in Pi sufficient solution were transferred to either Pi deficient or sufficient solution for defined treatment time as indicated before sampling. As showed in Figure 1, the root lengths of both cultivars did not show significantly change upon Pi deficiency when compared to those grown in Pi sufficient solution in 3 d treatment (Figure 1a). A sharp increase of root lengths was observed under Pi starvation after 7 d treatment and root lengths were significantly increased after 21 d of Pi deficiency treatment in both cultivars (Figure 1a). In contrast, Pi deficiency did not affect the shoot length over the entire observation period in both cultivars (Figure 1b). In terms of shoot lengths, there was no significant difference between the two cultivars regardless of Pi levels. However, the root lengths of Scout 66 were generally shorter than that of Atlas 66 in both Pi conditions, although the difference was not always remarkable.

**Figure 2:**
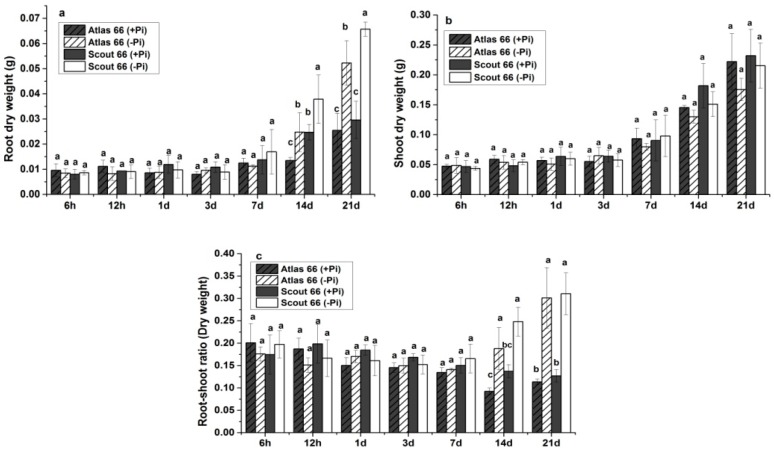
Root dry weight (**a**), shoot dry weight (**b**), and root-shoot dry weight (**c**) ratio of two wheat cultivars in different observed time points (6 h to 21 d) under the phosphate sufficient (250 µM) and deficient (0 µM) conditions. Error bars are SD, *n* = 6. Different small letters indicated significant differences (*p* ≤ 0.05) among the different treatment combinations within the same time point.

In agreement with the root length, as shown in Figure 2, the root biomass of both cultivars were not shown dramatically changed upon Pi deficiency under short- to mid-term treatments; while Pi deficiency led to a remarkable increase of root biomass in both cultivars under long-term treatment (Figure 2a). It is noticed that Scout 66 had higher root biomass than that of Atlas 66, particularly under Pi deficiency, at 14 d and beyond after transfer. By contrast, Pi deficiency generally resulted in a decrease of shoot biomass under long-term treatment, with about 21% and 7% shoot biomass being reduced in Atlas 66 and in Scout 66, respectively, when compared to Pi sufficient conditions (Figure 2b). Combining the root and the shoot biomass together, it is clear that Pi deficiency significantly increased the root-shoot ratio (DW) from 14 d onwards regardless of cultivars, but the difference of root-shoot ratio between cultivars was not significant (Figure 2c). Taken all the results together, Scout 66 can accumulate more biomass under Pi deficient conditions than Atlas 66, particularly under long-term Pi deficiency.

### 2.2. Scout 66 Produced Higher Root Volume and Had More Root Tip Number under Phosphate Deficiency

**Figure 3:**
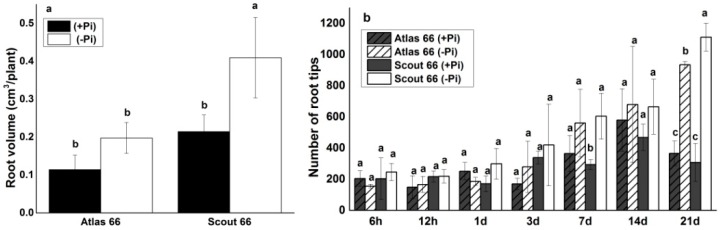
Root volume at 14 d time point (**a**), and root tips number (**b**) in different observed time points (6 h to 21 d) of two wheat cultivars in both phosphate sufficient (250 µM) and deficient (0 µM) conditions. Error bars are Standard Deviation (SD), *n* = 3. Different small letters indicated significant differences (*p* ≤ 0.05) among the different treatment combinations within the same time point.

It is believed that the root volume will be increased upon Pi deficiency to facilitate the Pi uptake from the soil. To further compare the Pi deficiency responses, the root volumes were measured in the two cultivars under long-term treatments. As shown in Figure 3, Pi deficiency led to an increase of root volume in both cultivars, but only in Scout 66 the increase was showed significant (Figure 3a). In addition, Scout 66 was showed to have bigger root volume than that of Atlas 66 at both Pi levels, especially under Pi deficiency (Figure 3a). To know whether the increased root volume is due to the increased lateral roots or not, we further counted the root tip numbers in both cultivars over a range of treatments. Similarly, it was not significantly changed in the root tip numbers under short- and mid-term Pi deficient treatments when compared to the Pi sufficiency (Figure 3b). However, with the extension of Pi deficiency, the root tip numbers increased, and a significant increase of root tip numbers was observed at 21 d after transfer in both cultivars, with Scout 66 having significantly higher root tip numbers than that of Atlas 66 under Pi deficient conditions (Figure 3b). Taken together, Scout 66 possesses significantly higher root volume and produces a greater number of root tips than Atlas 66 under Pi starvation.

### 2.3. Higher Total P and Soluble Phosphate Concentrations in Scout 66 under Phosphate Deficiency

**Figure 4:**
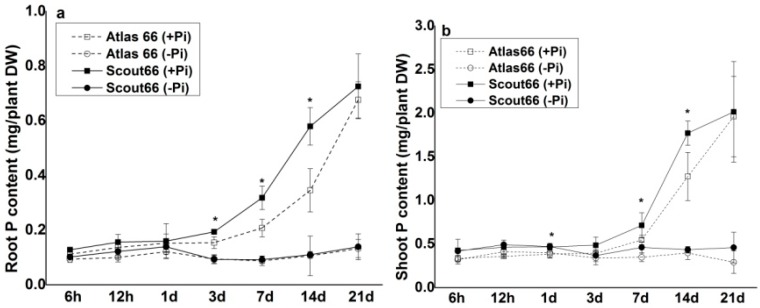
Root (**a**) and shoot (**b**) total phosphorus (P) of two wheat cultivars in different observed time points (6 h to 21 d) in both phosphate sufficient (250 µM) and deficient (0 µM) conditions. Error bars are SD, *n* = 3. Asterisk (*) indicated significant differences (*p* ≤ 0.05) between two cultivars under phosphate sufficient conditions at that time points.

To get more information about the Pi deficiency response of the two cultivars, we measured both P contents and Pi concentrations over a range of treatment periods. Overall, seedlings grown in Pi sufficient solution contain higher P content than that grown in Pi deficient solution, which is cultivar independent (Figure 4). In addition, in general the shoots, regardless of cultivars, contain higher P content than that of the roots (Figure 4). With the increasing of growth time, the P contents in both roots and shoots were shown increased sharply under Pi sufficient conditions; this increase is faster in the roots (after transfer of 3 d) than in the shoots (after transfer of 7 d) (Figure 4a,b). It is noteworthy that the increase magnitude of P contents in both roots and shoots was much higher in Scout 66 than that of in Atlas 66 under Pi-replete conditions (Figure 4a,b). By contrast, the P contents in both roots and shoots did not differ very much over the treatment periods under Pi deficient conditions, and no significant difference was observed either between the two cultivars (Figure 4a,b).

**Figure 5:**
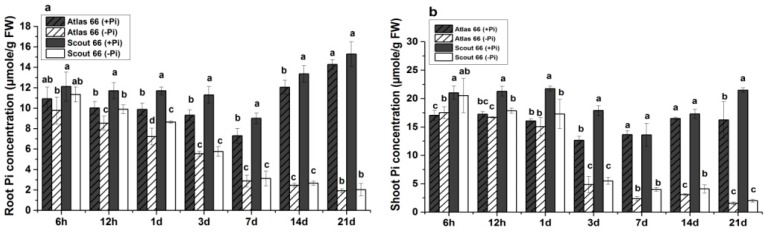
Root (**a**), and shoot (**b**) soluble phosphate (Pi) concentration of two wheat cultivars in different observed time points (6 h to 21 d) in both phosphate sufficient (250 µM) and deficient (0 µM) conditions. Error bars SD, *n* = 3. Different small letters indicated significant differences (*p* ≤ 0.05) among the different treatment combinations within the same time point.

It is believed that the cellular soluble Pi is associated with the metabolic activity, reflecting the plant’s fitness [42,43]. Thus, we further determined the Pi concentrations. In general, Pi deficiency led to a decrease of soluble Pi concentrations of both roots and shoots in both cultivars (Figure 5). Under Pi sufficient conditions, Scout 66 generally contained higher Pi concentrations in both roots and shoots than that of Atlas 66 (Figure 5a,b); while under Pi deficient conditions, Scout 66 roots contained higher Pi concentrations than that of Atlas 66 roots under short- and mid-term treatments (<3 d after transfer), but not under long-term treatment (Figure 5a). In the shoots, however, the Pi concentrations were observed decreased much earlier in Scout 66 than that in Atlas 66, with the Pi concentration being significantly decreased at 12 h, and 3 d after transfer in Scout 66 and Atlas 66, respectively (Figure 5b).

### 2.4. Scout 66 Contains Higher Iron (Fe) and Zinc (Zn) Contents in the Roots under Phosphate Deficiency

It has been reported that Pi deficiency leads to an increase of Fe content and alterations of other mineral elements [44,45,46,47]. In agreement with these reports, the contents of Fe were shown increased under Pi deficiency in the roots of both cultivars, particularly in the Scout 66, in which more than two fold-changes of Fe content were determined between Pi deficiency and Pi sufficiency. By contrast, the shoot Fe contents did not show significantly difference regardless of both treatments and cultivars (Table 1). In general, Pi deficiency leaded to a decrease of Zn content both in the roots and in the shoots of both cultivars. However, this decrease was significant only in the roots of Scout 66 (Table 1).

### 2.5. Scout 66 Can Maintain Higher Chlorophyll Content under Phosphate Deficiency

**Figure 6:**
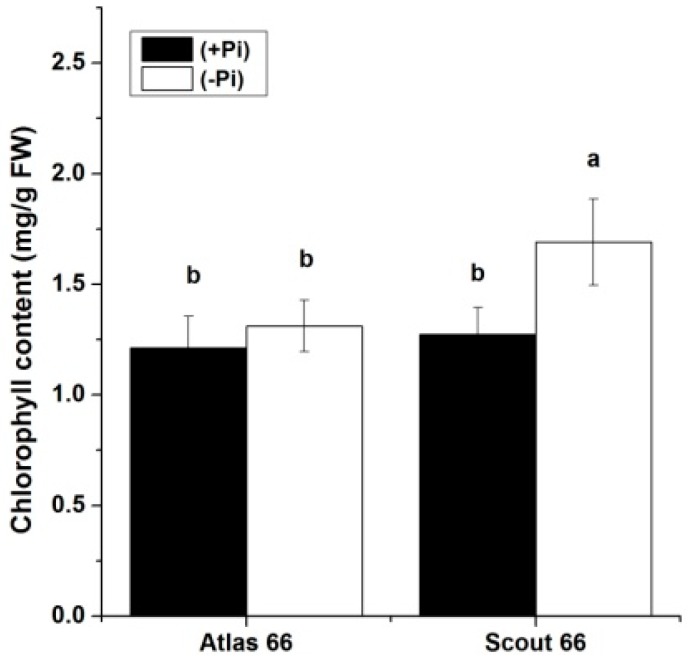
Chlorophyll content of two wheat cultivar at 21 d time point under phosphate sufficient (250 µM) and deficient (0 µM) conditions. Error bars are SD, *n* = 3, Different small letters indicated significant differences (*p* ≤ 0.05) among the different treatment combinations.

The leaf chlorophyll content is one of the major plant pigments and is usually used as a physiological index of plant fitness. The leaf chlorophyll contents in two cultivars were determined at 21 d after transfer under both Pi levels; and results showed that Pi deficiency led to an increase of chlorophyll contents in both cultivars (Figure 6). However, this increase was only significant in Scout 66, but not in Atlas 66.

### 2.6. The Molecular Responses of the Two Cultivars under Phosphate Deficiency

**Figure 7:**
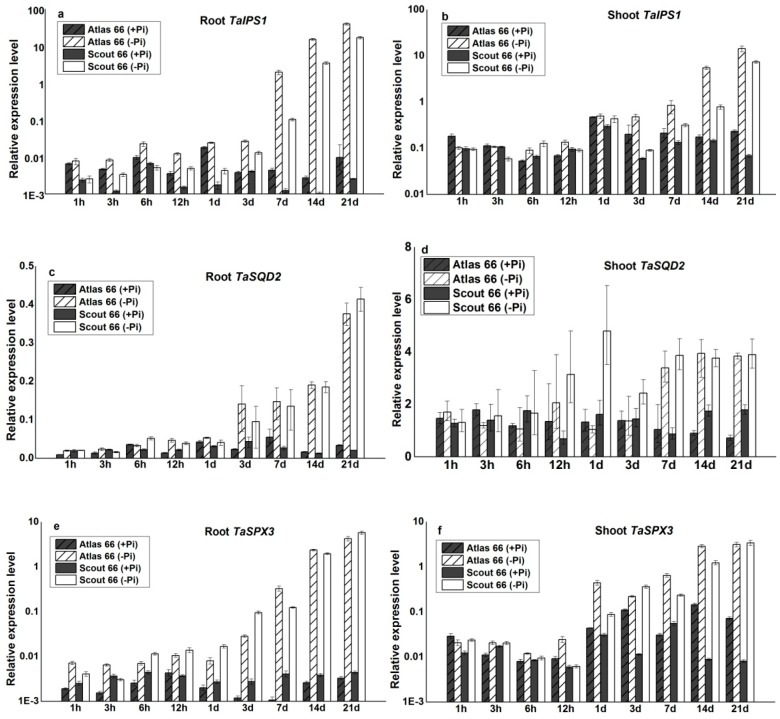
Relative mRNA expression levels of genes, *TaIPS1* in root (**a**) and in shoot (**b**), *TaSQD2* in root (**c**) and in shoot (**d**), and *TaSPX3* in root (**e**) and in shoot (**f**) of two wheat cultivars at 9 different time points (1 h to 21 d) in both phosphate sufficient (250 µM) and deficient (0 µM) conditions. Error bars are SD, *n* = 3.

To coordinate the Pi deficiency responses, plants have evolved sophisticated systems to sense the Pi-status and to regulate the expression of Pi starvation inducible (*PSI*) genes according to plant’s demand, both transcriptionally and post-transcriptionally [2,11,18,42]. To assess the molecular responses of the two cultivars to Pi deficiency, the expression of three known *PSI* marker genes, namely, a non-protein coding gene *IPS1* [48], *TaSQD2* involved in the biosynthesis of sulpholipids [49], and *TaSPX3* [50], were measured by using quantitative RT-PCR (qRT-PCR). Overall, the expression of these marker genes was induced and increased with the increasing periods of Pi starvation, reaching a maximum after 21 d of Pi starvation (Figure 7). Among them, *TaIPS1* showed the strongest induction both in the roots and in the shoots in both cultivars (Figure 7a,b). It was noticed that the steady-state abundance of *TaIPS1* was lower in the roots of Scout 66 than that in the Atlas 66 under Pi sufficiency, while the abundance is comparable in the shoots in the two cultivars (Figure 7a,b). In addition, the steady-state abundance of *TaIPS1* was relatively higher in the shoots than in the roots under Pi sufficiency regardless of cultivars.

Compared to the early response of *TaIPS1*, the induction of *TaSQD2* was later, but a remarkable increase of *TaSQD2* was observed in the roots at 3 d of Pi starvation and reached a maximum at 21 d after initiating the Pi treatment (Figure 7c). Moreover, this response was comparable in the roots between two cultivars (Figure 7c). Similarly, the induction of *TaSQD2* was later in the shoots, but earlier in the roots, being upregulated after 12 h of Pi starvation, but this only occurred in Scout 66 not in Atlas 66 (Figure 7d). Under long-term Pi deficient conditions, the expression of *TaSQD2* was strongly induced in both cultivars (Figure 7d).

Similar to *TaIPS1*, overall, the steady-state abundance of *TaSPX3* was very low in the roots under Pi sufficiency, regardless of cultivars. Nevertheless, its expression was strongly induced upon Pi deficiency and this induction occurred as early as 1 h of Pi starvation in both cultivars (Figure 7e), reaching a maximum after 21 d of Pi starvation. In general, the magnitude of induction was comparably higher in Atlas 66 than in Scout 66 (Figure 7e). In the shoots, however, the inductions of *TaSPX3* were later than that of in the roots, being upregulated significantly after 1 d of Pi starvation, reaching a maximum induction after 21 d of Pi starvation; and the induction extents were stronger in Scout 66 than that of in Atlas 66 under long-term Pi starvation (Figure 7f).

## 3. Discussion

In modern agriculture systems, the demand for P-fertilizer is increasing and indispensable for crop productivity, which is becoming urgent to ensure food security under negative impacts of climate change and growing population in the world. Wheat is one of the main crops and sometimes the only food source in some areas. Maintaining and increasing wheat production is therefore critically important in the world [6]. However, quite high percentage of wheat is produced in acidic soils with low Pi levels, resulting in low wheat yields. Enhancing wheat productivity under low Pi conditions is thus urgent and important. It has been reported that different species and varieties exhibit contrasting tolerance to low Pi stress [51,52]. In the present study, with the attempt to explore more gene resources involved in the low Pi adaptation in wheat, we chose to compare the physiological and molecular responses of two wheat cultivars, contrasting in Al tolerance, to Pi deficiency. Initially, the reason why to choose these two cultivars is based on this suspicion that Al-tolerant cultivar might have better performance than that of Al-sensitive one. Because under acidic soils, in addition to the negative impacts of low Pi stress on plant growth, Al toxicity, particularly under pH below 4.5, is a must-be considering factor that hinders plant growth and development [37]. To exclude the side effects of Al toxicity, the wheat seedlings were cultured in a hydroponic system under controlled conditions and the only variable is the Pi concentration as mentioned in the materials and methods.

Unexpectedly, under Pi deficiency, the Al-tolerant cultivar Atlas 66 did not grow better than that of Al-sensitive cultivar Scout 66, in terms of root and shoot biomass (Figure 2) and chlorophyll content (Figure 6). The better performance of Scout 66 under Pi deficiency is likely because of its larger root volume and root tip numbers (Figure 3), rather than root lengths (Figure 1). It is well established that root architecture plays an important role in Pi uptake from the soil and is discussed to be a target for Pi-efficiency breeding [5,6,7,8,10,14]. Indeed, transgenic rice lines with high expression of *PSTOL1* exhibited larger root system to confer tolerance of Pi deficiency, leading to superior performance [51]. A similar finding was also reported in Arabidopsis [52]. A larger root system not only enhances Pi acquisition, but also facilitates the uptake of water and other nutrients, such as nitrogen, which confers the plants to have better fitness under abiotic stresses. Indeed, we found that scout 66 contains higher chlorophyll content under long-term Pi starvation (Figure 6), which could be due to the larger root dry biomass caused by larger root volume (Figure 3). Although whether the higher content of chlorophyll is due to high nitrogen content in the shoots remains elusive, Scout 66 is indeed more tolerant to nitrogen deficiency than that of Atlas 66, with being much delayed yellowing upon nitrogen starvation (in preparation). The transcript factors or regulatory factors, such as kinase [53], controlling the root volume and tip numbers in Scout 66 awaits future study. Taken together, from morphological traits, it seemed that Scout 66 shows better performance in response to nutritional deficiencies than that of Atlas 66.

The higher Pi concentration might represent better metabolic adaptations to Pi deficiency [43]. In this study, Scout 66 was found to contain both high total P and soluble Pi under Pi deficiency (Figure 4 and Figure 5), which might suggest that Scout 66 possess more active metabolic activity to adapt to Pi deficiency, leading to the larger dry biomass and root volume. In the future, comparative metabolomics of the two cultivars to Pi starvation response would provide insight into the molecular mechanism why Scout 66 performs better upon Pi deficiency.

It is established that both Fe and Zn are essential micronutrients for plant growth and development and their homeostasis is altered upon Pi deficiency [45,54,55,56]. Previous studies have reported that Pi starvation promotes metal accumulation in plants, mainly aluminum, and Fe [45,55,56,57]. *PHR1* has been documented to be involved in the interactions between Fe and Pi, as well as the interaction between Pi and Zn [45,55,56]. Long-term Pi deficiency leads to Zn over-accumulation in Arabidopsis [44]. In line with this, our results showed, at long-term Pi deficiency, that overall increased Fe contents in the roots were observed (Table 1). However, only in the Scout 66, this increase was significant (Table 1). Future study will address whether the increased Fe content in the Pi deficiency Scout 66 roots is due to the formed iron plaque (IP) in the root system upon Pi deficiency, or due to the more Fe uptake. By contrast, under our conditions, in general, long-term Pi deficiency leads to a decrease of Zn content in both roots and shoots, regardless of cultivars (Table 1), which is controversy to previous study in Arabidopsis [44], but is in agreement with the study in wheat [58]. A significant decrease of Zn content in the Scout 66 roots under long-term Pi deficiency might be due to the pronounced increase of Fe content (Table 1), which is agreement with previous observation that excess Fe will cause Zn deficiency [45]. In the future, the interactions between Pi and Fe, Pi and Zn, and Fe and Zn are worthy of further studies in wheat.

Over the past decade, the molecular responses to Pi deficiency have been extensively explored and subsets of core Pi-responsive genes have been identified in model plants Arabidopsis, as well as in rice [11]. Nevertheless, due to the high genome complex and limited genome information available, as well as the low efficiency of gene transformation, the wheat molecular responses to Pi deficiency remain fragmentary [6]. In this study, although tried our best, we failed to obtain consistent results on the expression of high affinity Pi transporter genes. However, we succeeded to determine the expression of three well-known Pi responsive genes by means of qRT-PCR (Figure 7). Because *IPS1* is reported robustly upregulated upon Pi starvation and suggested to be involved in the early Pi deficiency-mediated signal transduction [2]. We thus first analyzed the expression patterns of *IPS1* in wheat, *TaIPS1*. The expression of *TaIPS1* was induced very early upon Pi starvation, but the expression patterns were comparable in both cultivars under Pi deficiency (Figure 7). We thus suggest that *TaIPS1* is a good Pi-responsive marker gene in wheat, but may be not suitable for indicating the differential response in different cultivars. This could be applied to another Pi-responsive marker gene *TaSQD2*, although whose induction by Pi deficiency is much later than that *TaIPS1*. In contrast to the later induction of *TaSQD2*, *TaSPX3* was an early-response gene upon Pi deficiency (Figure 7). The *SPX* gene family has been documented to play a major role in controlling the expression of *PSI* genes [20,27,34,35,59], the early-response and strong induction of *TaSPX3* implicated that this regulator might play an important role in wheat, although we could not rule out the possibility that other *SPX* genes are also critical for wheat to deal with Pi deficiency. The induction extent of *TaSPX3* in the roots is higher in Atlas 66, but in the shoots is higher in Scout 66 (Figure 7). This contrasting induction in the two cultivars might imply the differential responses to Pi starvation. In the roots, Atlas 66 produced smaller root systems than that of Scout 66, which might lead to Atlas 66 more sensitive to Pi starvation, since larger root systems can enhance the uptake of nutrients. While in the shoots, Scout 66 contains higher chlorophyll than that of Atlas 66, which probably leads Scout 66 to have stronger photosynthesis. To maintain stronger photosynthesis, more Pi is required, which is transported from the roots to the shoots, leading to a stronger induction of *TaSPX3* and subsequently the downstream gene expression. This explanation can be supported by the fact that Scout 66 contains higher Pi concentration in the shoots under Pi deficiency (Figure 5). In addition, Scout 66 accumulated more root dry biomass, which requires more sugar produced in the shoots to transport to the roots, further supporting our hypothesis. Nevertheless, this hypothesis remains to be further verified by means of transcriptome coupled with biochemical analysis.

## 4. Materials and Methods

### 4.1. Plant Materials and Growth Conditions

Wheat (*Tritium aestivum* L.) seeds were surface sterilized with 10% (*v*/*v*) H_2_O_2_ for 30 min and rinsed five times with distilled water, and then soaked in distilled water for another two hours (h). The seeds were germinated in a petri dish with filter paper saturated with distilled water in darkness at 26 °C for 3 days. Followed by, the seedlings with similar size were transferred into a plastic box containing 7.5 L Modified Hoagland solution (1/4th strength) for about 3 weeks until the third leaves were fully developed. Next, half of the wheat plants were treated with nutrient solution without P (0 µM Pi, −Pi) and the rest of the wheat plants were remained in the nutrient solution with sufficient P (250 µM Pi, +Pi). The Modified Hoagland solution (1/4th strength) was described as follows: Ca(NO_3_)_2_·4H_2_O (2 mM), MgSO_4_·7H_2_O (650 μM), KH_2_PO_4_ (250 μM), K_2_SO_4_ (750 μM), MnSO_4_·H_2_O (10 μM), CuSO_4_·5H_2_O(0.1 μM), ZnSO_4_·7H_2_O (1 μM), H_3_BO_3_ (1 μM), (NH_4_)_6_MoO_24_·4H_2_O (0.05 μM), KCl (100 μM), Fe-EDTA (40 μM). The nutrient solution without Pi was supplemented with KCl (250 µM) to make an equal K^+^ supply. The nutrient solution was exchanged every 3 days and the pH was adjusted to 6.0 with NaOH. All experiments were conducted in a controlled environmental condition maintained at 26 °C with 65% relative humidity. Photoperiod was adjusted to 14 h/10 h light/dark condition with a light intensity of 200 µmol photon m^−2^·s^−1^.

### 4.2. Root and Shoot Length, Biomass, Root Volume and Root Tip Numbers

The samples were collected from short (6 h to 1 d), mid (1 d to 7 d) and long time periods (7 d to 21 d) under both Pi levels. To minimize the circadian effect, sample collection was performed at the same time (10 am) of the day. The roots were rinsed several times with distilled water to remove the surface ions and blotted dry with tissue papers. After measuring root and shoot length, the samples were dried at 72 °C in the oven for 60 h.

To measure root volume and tip numbers, the harvested roots were dispersed in a transparent tray with water, and then scanned with a root system scanner (EPSON Expression 1600, Seiko EPSON Corp., Nagano, Japan). The scanned images were analyzed by WinRHIZO image analysis system (WIN MAC, Regent Instruments Inc., Quebec, QC, Canada, http://www.regentinstruments.com/).

### 4.3. Measurement of Total Phosphorus and Soluble Phosphate Concentration

To determine the total P content, the dried root and shoot samples were digested with 2 mL H_2_SO_4_ (2 h at 150 °C and 3 h at 180 °C), thereafter H_2_O_2_ was added to make them properly digest. Total P content was determined using molybdenum blue-colorimetric method [60]. To measure soluble Pi, the root and shoot (around 50 mg, fresh weight) were homogenized with extraction buffer (10 mM Tris, 1 mM EDTA, 100 mM NaCl, 1 mM β-mercaptoethanol, and 1 mM PMSF) and the Pi concentration was subsequently determined as described in [61].

### 4.4. Measurement of Iron and Zinc Concentration

Root and shoot samples were harvested at 21 d time point and used to determine the iron and zinc concentration. The roots were washed with 2 mM CaSO_4_ for 10 min and 10 mM EDTA for another 10 min to remove the surface ions. The dried samples were digested with HNO_3_ (2 mL), and the Fe and Zn concentrations were determined by ICP-MS (Optima 8000 ICP-OES Spectrometer, PerkinElmer, Inc., Waltham, MA, USA).

### 4.5. Measurement of Chlorophyll Content

For chlorophyll content analysis, the fresh leaves collected at 21 d (0.2 g) were placed into vials containing 10 mL of 80% (*v*/*v*) acetone. The vials were placed in a dark place at room temperature overnight to ensure a complete leaching of the pigments. The leaf total chlorophyll content was determined according to the method by Arnon [62]. The absorbance values for the chlorophyll a and chlorophyll b were recorded at the wavelength of 663 nm and 646 nm, respectively and subsequently determined chlorophyll content.

### 4.6. Phosphate Induced Marker Genes Expression Analysis by qRT-PCR

The fresh roots and shoots were collected in the scheduled time point (for the marker gene expression analysis, 1 h and 3 h time points were included), and immediately kept in liquid nitrogen and stored at −80 °C for further analysis. For RNAs extraction, fresh tissues were grinded with a mortar and pestle and total RNAs were extracted using TRIzol reagent (Invitrogen, Karlsruhe, Germany) according to the manufacturer’s instructions. RNA concentration was determined with NanoDrops (Thermo Scientific, NanoDrop 2000 Spectrophotomer, Wilmington, DE, USA) and its integrity was verified through agarose gel electrophoresis. 1 µg of total RNA from each sample was converted into cDNAs using Prime Script reverse transcriptase (Invitrogen, Karlsruhe, Germany). Quantitative real time PCR (qRT-PCR) was applied to determine the gene expression level using the SYBR green PCR Master Mix (Takara Bio Inc., Kusatsu, Japan). The program was described as follows: Pre-denaturation at 95 °C for 30s, 40 cycles of denaturation at 95 °C for 5 s and annealing at 60 °C for 30 s, followed by melt-curve analysis (60 °C–95 °C, 0.5 °C increments for 5s). The relative expression levels of different studied genes were calculated according to the 2^−Δ*C*t^ method [63]. The primers used in this study were listed in the Supplementary Table S1. The wheat *TaAPT1* (Adenine phosphoribosyltransferase 1) gene was used as a house keeping gene.

### 4.7. Statistical Analysis

All of the data presented here were mean values for each treatment. Two-way analysis of variance (ANOVA) was carried out between cultivars and *p* under control and treatments and followed by the least significant difference (LSD) multiple range test (*p* < 0.05) using SPSS version 22 statistical software (IBM Corp., Armonk, NY, USA).

## 5. Conclusions

In this study, two genotypes contrasting in Al-tolerance were comparatively analyzed, focusing on the morphological, physiological and molecular characteristics under Pi deficient conditions. It was concluded that the Al-sensitive cultivar, Scout 66, exhibited better performance under Pi deficient conditions compared to Atlas 66. With the common wheat genome sequencing completed and more genome information available, it provides us more chance to uncover the molecular mechanism by which Scout 66 performs better under Pi deficiency than Atlas 66 in the near future. Combined the Al—tolerance related genes with the Pi-efficiency genes, it is possible to develop Pi-efficiency wheat cultivars that have good fitness on acidic soils.

## Figures and Tables

**Table 1:** Iron and zinc content in root and shoot in two wheat cultivars under phosphate sufficient (250 µM) and deficient (0 µM) conditions.

Cultivars	Phosphate Treatment	Fe (mg/g DW)		Zn (µg/g DW)	
		Root	Shoot	Root	Shoot
Atlas 66	(+Pi)	7.08 ± 2.64 ^b^	0.13 ± 0.004 ^a^	128.5 ± 5.0 ^b^	65.6 ± 17.1 ^a^
	(−Pi)	8.31 ± 1.51 ^ab^	0.12 ± 0.024 ^a^	106.5 ± 19.5 ^b^	49.7 ± 8.7 ^a^
Scout 66	(+Pi)	5.62 ± 1.45 ^b^	0.10 ± 0.044 ^a^	189.2 ± 39.0 ^a^	68.2 ± 9.8 ^a^
	(−Pi)	12.41 ± 4.25 ^a^	0.12 ± 0.007 ^a^	114.5 ± 1.5 ^b^	60.6 ± 4.3 ^a^

Seedlings were grown in both Pi levels at 21 d time point. Different small letters indicated significant differences (*p* ≤ 0.05) among the different treatment combinations, (±) are Standard Deviation (SD), *n* = 3.

## Supplementary Materials

Supplementary materials can be found at http://www.mdpi.com/1422-0067/19/10/2964/s1.
