# Supplementary material for: Can Aluminum Tolerant Wheat Cultivar Perform Better under Phosphate Deficient Conditions?
Source: Int J Mol Sci. 2018 Sep 28;19(10):2964. doi: 10.3390/ijms19102964 (PMC6213158; doi:10.3390/ijms19102964)
Supplement: Supplementary file 1 [file ijms-19-02964-s001.pdf]

Supplementary Materials

Can Aluminum Tolerant Wheat Cultivar Perform Better under Phosphate Deficient Conditions?

Table S1: List of primers use for quantitative RT-PCR (qRT-PCR).

Genes	Forward primer sequence (5′–3′)	Reverse primer sequence (5′–3′)	Accession number
TaIPS1	GACACTGAAGACTCGCACCA	CGGCGACTTCTCACCTCTAC	EU753151.1
TaSQD2	TGCCAGTGGAGATGTGTTTGT GAT	AAGCTGGTCTTTCCTTCCTGAT	AK333217.1
TaSPX3	GTGGAAGGACGAGTTCCTGAG C	TCCCGGTGTGTGATGATGAAGAA	AK333013.1
TaAPT1 *(Reference gene)*	CGAATCAGTATGAAACAAGTT GTGACTCTT	TCCCAAACAGTTCCAGAAGGACAAAT	U22442.1
